# Severe paraneoplastic hypereosinophilia in metastatic renal cell carcinoma

**DOI:** 10.1186/1471-2490-12-7

**Published:** 2012-03-21

**Authors:** Tilman Todenhöfer, Stefan Wirths, Claus Hann von Weyhern, Stefan Heckl, Marius Horger, Joerg Hennenlotter, Arnulf Stenzl, Lothar Kanz, Christian Schwentner

**Affiliations:** 1 Department of Urology, University Hospital Tuebingen, Tuebingen, Germany; 2 Department of Internal Medicine, Oncology, Hematology, University Hospital Tuebingen, Tuebingen, Germany; 3 Department of Pathology, University Hospital Tuebingen, Tuebingen, Germany; 4 Department of Neuroradiology, University Hospital Tuebingen, Tuebingen, Germany; 5 Department of Radiology, University Hospital Tuebingen, Tuebingen, Germany; 6 Department of Urology, Eberhard-Karls University, Hoppe-Seyler Strasse 3, D-72076 Tübingen, Germany

**Keywords:** Paraneoplastic, Hypereosinophilia, Leukocytosis, Renal cell carcinoma, Leukemoid reaction, Encephalopathy

## Abstract

**Background:**

Renal cell carcinoma can cause various paraneoplastic syndromes including metabolic and hematologic disturbances. Paraneoplastic hypereosinophilia has been reported in a variety of hematologic and solid tumors. We present the first case in the literature of severe paraneoplastic hypereosinophilia in a patient with renal cell carcinoma.

**Case presentation:**

A 46 year-old patient patient with a history of significant weight loss, reduced general state of health and coughing underwent radical nephrectomy for metastasized renal cell carcinoma. Three weeks after surgery, the patient presented with excessive peripheral hypereosinophilia leading to profound neurological symptoms due to cerebral microinfarction. Systemic treatment with prednisolone, hydroxyurea, vincristine, cytarabine, temsirolimus and sunitinib led to reduction of peripheral eosinophils but could not prevent rapid disease progression of the patient. At time of severe leukocytosis, a considerable increase of cytokines associated with hypereosinophilia was measurable.

**Conclusions:**

Paraneoplastic hypereosinophilia in patients with renal cell carcinoma might indicate poor prognosis and rapid disease progression. Myelosuppressive therapy is required in symptomatic patients.

## Background

Renal cell carcinoma can cause various paraneoplastic syndromes such as hypercalcemia, hypertension and ectopic hormone production [1]. Renal cell carcinoma can also provoke hematologic disturbances such as polycythemia due to an increased production of erythropoietin [2]. Hypereosinophilia has been reported as a paraneoplastic syndrome in several solid and hematological malignancies. We report the first case of severe paraneoplastic hypereosinophilia with cerebral infarction in a patient with metastatic renal cell carcinoma.

## Case Presentation

### Case presentation and management

**Figure 1:**
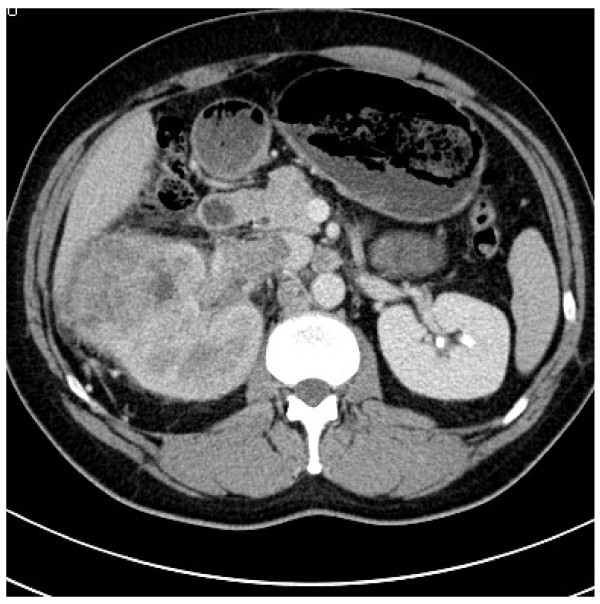
**Preoperative imaging showing a hypervascularized renal tumor with a level I tumor thrombus in the vena cava**.

A 46 year-old patient with no relevant diseases in past medical history had a history of significant weight loss, reduced general state of health and coughing. A whole body CT revealed a hypervascularized renal tumor with a level I tumor thrombus [3] (Figure 1) and multiple pulmonary lesions. At the time of primary diagnosis, blood analysis showed a WBC of 19,550/μl (4,000-7,000/μl) with 16% (0-6%) of eosinophilic granulocytes. The patient was admitted to our hospital 7 days after primary diagnosis (day 7) for radical nephrectomy, partial hepatectomy and reconstruction of the inferior vena cava. The intra- and postoperative course was uneventful. Histological examination showed clear cell renal cell carcinoma with sarcomatoid components (Tumor stage: pT4, pNx, M1, L0, V1, Rx, G3). According the MSKCC criteria, the patient was intermediate risk at time of diagnosis [4]. In an intersciplinary tumor board, the patient was recommended to begin with oral sunitinib. The patient was discharged from hospital 7 days after surgery (day 14) with a WBC of 14,360/μl. On day 29 the patient again presented at our hospital in a reduced general condition, with fever of 38.4°C, tachycardia. Laboratory examination demonstrated leukocytosis of 37 × 10^3^/μl with 34.2% of eosinophilic granulocytes. Ultrasound revealed a partially liquid mass with 10 × 5 cm of size, which was suspicious of abscess formation, confirmed by computed tomography. The liquid mass was subsequently drained and 700 mL of serous fluid could be evacuated. Cultures of the fluid remained sterile. The patient was treated with intravenous antibiotics (vancomycine, tazobactam/piperacilline, and ciprofloxacin). However, leukocytes and eosinophilic granulocytes increased further despite antibiotic therapy and drainage.

**Figure 2:**
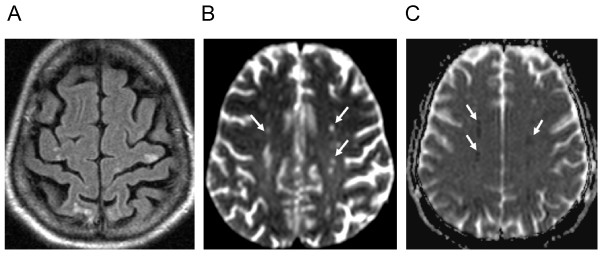
**A: axial FLAIR image shows multifocal cortical and subcortical hyperintense lesions (arrows) presumed to be of embolic origin**. B and 2 C: axial diffusion-weighted image-DWI (b-value, 1,000 s/mm^2^) (Figure 2B) and corresponding apparent diffusion coefficient (ADC) map demonstrate marked water diffusion restriction in acute embolic ischemia (arrows). Note the drop in signal intensity on ADC-map (Figure 2C, arrows).

**Figure 3:**
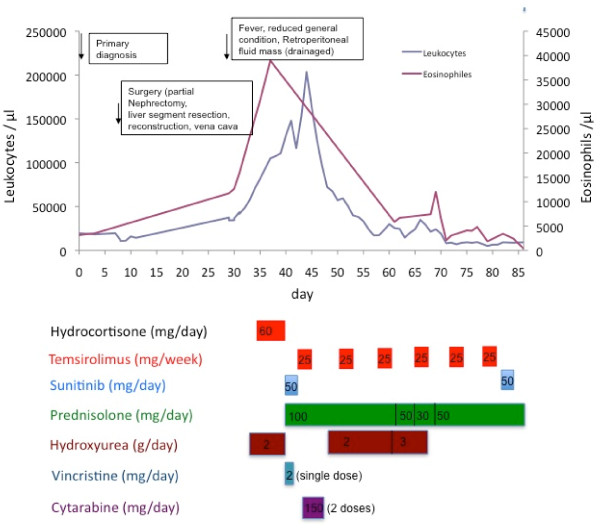
**Leukocytes and eosinophilic granulocytes count**. Lower bars show doses of applicated drugs.

**Figure 4:**
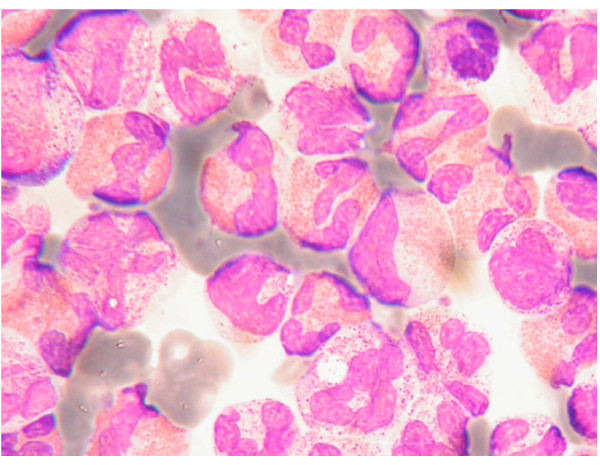
**Peripheral blood smear hypereosinophilia with hypersegmented forms**.

On day 33, the patient presented with a weakness of the left arm. Multiple fresh embolic lesions were detected by MRI (magnetic resonance tomography) in the parietal, temporal and occipital lobes bilaterally (Figure 2). Furthermore a small infarction was detected in the cerebellum which led to the suspicion of cardiac emboli. Transthoracal echocardiography showed a profoundly decreased function of the left ventricle with inferior hypokinesia, a low-grade mitral valve insufficiency and no evidence of structures characteristic for endocarditis. The corresponding electrocardiogram showed a sinus tachycardia with ventricular extrasystoles (Lown classification 2). Ultrasound of extracranial vessels did not show significant stenosis. As at this point leukocytes had further increased up to 57,390/μl with 37.7% of eosinophilic granulocytes (Figures 3 and 4).

Several tests were performed to rule out non-cancer causes of hypereosinophilia such as parasitic infections, allergy (including determination of serum mast cell tryptase), hypereosinophilic syndrome due to FIP1L1/PDGFR receptor mutations and aberrant lymphocytes (no CD4-positive CD3-negative lymphocytes characteristic for lymphoproliferative hypereosinophilic syndrome), which remained all negative. Consequently, the patient was submitted to the department of medical oncology and received steroids and cytoreductive therapy with hydroxyurea. Doses of hydroxyurea and prednisone were increased stepwise up to 3 g/day and 100 mg/day, respectively. Moreover, the patient received vincristine once (2 mg) and 2 doses of cytarabine (2 × 150 mg/24 h) as cytoreductive therapy of symptomatic hypereosinophilia (Figure 3).

**Figure 5:**
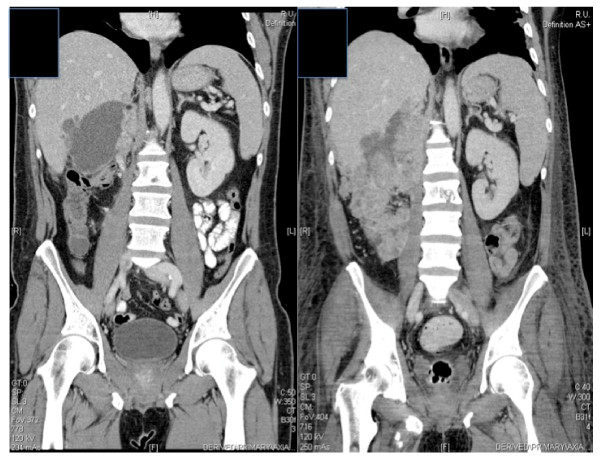
**Progression of retroperitoneal tumor mass after radical nephrectomy within 4 weeks (coronal CT-images)**.

A CT performed 2 weeks after the drainage of the fluid collection showed a massive progression of the retroperitoneal tumor mass in the surgical bed (Figure 5). Progress of the pleural and pulmonary metastases could be observed as well. Systemic therapy with temsirolimus (25 mg/once a week) was initiated, as at this point, swallowing difficulties (leading to parental nutrition) did not permit oral therapy with sunitinib. This led to a short-term response of pulmonary and pleural manifestations (according to RECIST). The retroperitoneal tumor mass also responded to temsirolimus with tumor necrosis. However, a CT performed one month after initiation of temsirolimus therapy revealed a progressive disease of local relapse with hepatic, pancreatic and diaphragmatic invasion.

Administration of antiproliferative drugs for treatment of hypereosinophilia (cytarabine, hydroxyurea and vincristin) and renal cell carcinoma led to significant reduction of leukocytes and eosinophils as well as pancytopenia (Figure 3).

We performed a multiplex cytokine assay based on Luminex^R ^technology (Progen^R^) to determine levels of multiple cytokines of serums drawn on day 44 (> 200.000 leukocytes/μl) and day 74 (8,580 leukocytes/μl). On day 44, we found severely increased concentrations of multiple cytokines and their receptors including bFGF, G-CSF, GM-CSF, HGF, IL2-RA, MCP-1 and MIP-1b. Results are shown in Table 1.

**Table 1:** Levels of multiple cytokines at time of excessive leukocytosis (day 44) and after administration of cytoreductive drugs) (day 74) measured by ultiplex cytokine testing (Progen)

Cytokine	Unit	Reference	Day 44	Day 74
EGF	pg/ml	-780	201	15,8

Eotaxin	pg/ml	175.8 ± 49.3	20,4	11,6

**FGF-Basic**	pg/ml	1,5-6,0	**40,8**	**23,5**

**G-CSF**	pg/ml	27.34 ± 8.00	**93,5**	**< LOW >**

**GM-CSF**	pg/ml	-2,3	**40,6**	**19,8**

**HGF**	pg/ml	120 ± 120	**< HIGH >**	**4480**

IFN-a	pg/ml	16.8 ± 6.59	25,3	< LOW >

IFNy	pg/ml		< LOW >	< LOW >

IL-10	pg/ml	9.2 ± 1.5	19,3	15,8

IL-12 p40/p70	pg/ml	171.1 ± 6,25	104	52,6

IL-13	pg/ml	25.5 ± 2.94	35,7	< LOW >

IL-15	pg/ml	16.2 ± 4.0	< LOW >	45,2

IL-17	pg/ml	0-127	28,8	23,6

IL-1b	pg/ml	40.2 ± 8.78	25,7	12,5

**IL-1RA**	pg/ml	189 ± 22	**979**	**241**

IL-2	pg/ml	2.4 ± 0.8	9,16	6,01

**IL-2R**	pg/ml	426.5 ± 22.4	**12300**	**18300**

**IL-4**	pg/ml	3.34 ± 0.84	**35,8**	**< LOW >**

IL-5	pg/ml		< LOW >	< LOW >

**IL-6**	pg/ml	22.8 ± 7	**330**	**299**

IL-7	pg/ml		66,4	47

**IL-8**	pg/ml	9.56 ± 0.4	**652**	**111**

**IP-10**	pg/ml	4.5-27.1	**73,6**	**27,3**

**MCP-1**	pg/ml	173.2 ± 15.04	**1110**	**377**

MIG	pg/ml	22.1-52.4	< LOW >	< LOW >

MIP-1a	pg/ml	88.1 ± 14.31	179	45,8

**MIP-1b**	pg/ml	135.1 ± 29.22	**2640**	**278**

RANTES	pg/ml	1100-4360	3750	664

TNFa	pg/ml	34.32 ± 11.46	9,21	5,81

VEGF	pg/ml	76.6 ± 6.07	65,5	24,1

Low = concentration below detection limit, High = above detection limit. Reference values were obtained from [5-7]

As swallowing difficulties disappeared, according to his will the patient was set on sunitinib 50 mg/d two months after nephrectomy. The patient could be discharged from hospital in reduced general condition 11 weeks after nephrectomy. Due to further disease progression, the patient died 4 months after primary diagnosis.

## Discussion

**Figure 6:**
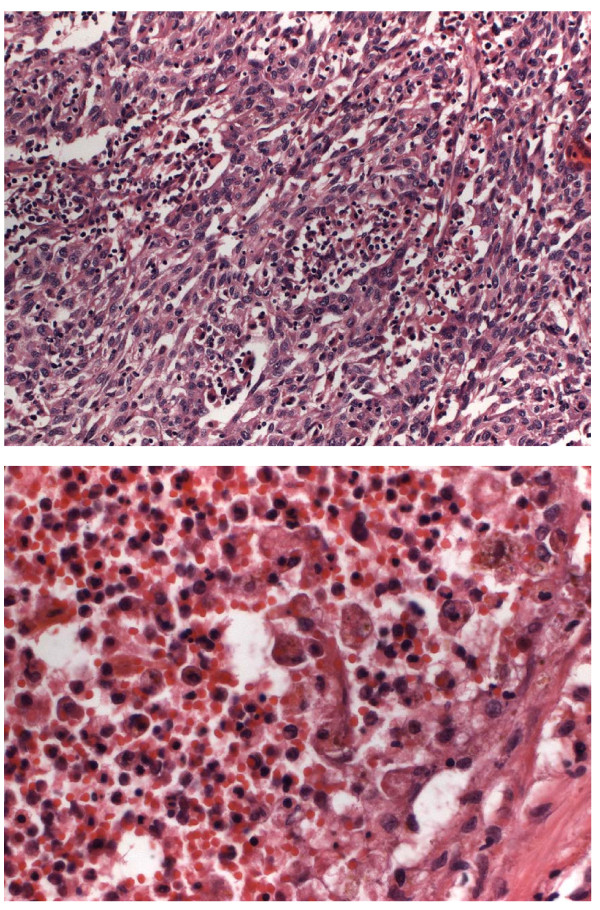
**A and B: Resection specimen of the right kidney and adrenal gland reveals a poorly, in parts sarcomatoid differentiated renal carcinoma**. Gross examination shows infiltration of the adenal gland, renal plevis and macroscopic vascular invasion. A: (H&E, 200×) Spindle like shaped tumor cells with small eosiniphilic cytoplasmn and pleomophic nuclei with eosinophilic nucleoli. Interspersed small amounts of histiocytes and small lymphocytes. The amount of eosinophils is not elevated in viable tumor areas. B: (H&E, 400×): Areas of tumor necrosis withs rims of histiocytes and increased number of eosinophils.

Hypereosinophilia is most commonly associated with allergy and parasitic infections. Furthermore, several drugs, pulmonary and gastrointestinal diseases have been identified as important causes for hypereosinophilia [8,9]. Within recent years, a couple of molecular alterations have been identified to account for malignant hypereosinophilic syndromes including rearrangements of the PDGFR receptor [8]. A mild to moderate increase (> 500/μl to < 1,500/μl) of eosinophils can be found in up to 5% of patients with malignancies [10]. Severe peripheral hypereosinophilia (> 5,000/μl) has been described in a variety of hematologic and solid tumors including gastrointestinal tumors [11,12], bronchial carcinoma [10], sarcomas [13] and prostate cancer [14]. Several cytokines produced by the primary tumor have been identified to account for increased production of eosinophilic granulocytes in the bone marrow including interleukin-3, interleukin-5 and GM-CSF (granulocytes macrophages stimulating factor) [12,15,16]. Other mechanisms for hypereosinophilia in patients with malignancy include an eosinophilotactic response due to necrosis in the tumor and increased production of eosinophils due to tumor cell dissemination in the bone marrow [9]. In the case presented here, we found increase of multiple cytokines at the time of massive leukocytosis and hypereosinophilia. G-CSF and GM-CSF which may be secreted by tumor cells and induce production of eosinophils [17,18] were significantly elevated. Soluble receptor of interleukin 2 (IL2-RA) has been shown to be an important mediator of autocrine and paracrine regulation of eosinophils [19]. MIP-1a which is secreted by eosinophils and induces further leukocyte activation was also significantly elevated [20]. Both, elevation of interleukin 8 and MCP-1, may be explained by TNF-mediated activation of eosinophils [21]. All these cytokines were significantly lower at time of normal leukocyte count. However, it is not possible to define the exact mechanism of hypereosinophilia in this patient, as the source of the cytokines remained undefined and mediators causing hypereosinophilia may also be induced by eosinophils themselves. The tumor presented with extensive necrotic areas showing massive infiltration of eosinophils (Figure 6). Tumor necrosis has been discussed as a factor promoting tumor related hypereosinophila [22]. However, as a steady increase of leukocytes was observed after resection of the tumor with necrotic areas, tumor necrosis cannot be regarded as the only promoter of increasing eosinophils in the present case and other sources for cytokines promoting hypereosinophilia are probable.

Paraneolastic eosinophilia is usually mild without any clinical symptoms, but absolute counts may occasionally exceed 25,000/μl and may cause end-organ damage. In a few reported cases of paraneoplastic hypereosinophilia, neurological symptoms occurred due to thromboembolic events with multiple infarctions [23,24,14]. Significantly reduced left ventricular function in our patient may be interpreted as end-organ damage as well.

Several studies have shown that paraneoplastic eosinophilia is a poor prognostic sign and indicates metastatic and extensive disease [25,26]. In a series of 36 cases with paraneoplastic hypereosinophilia, 32 patients had metastatic disease [27]. The patient reported here had a disseminated disease with pulmonary and bone metastases and presented with extraordinary rapid disease progression with poor response to surgical and systemic treatment. In previously reported cases, peripheral eosinophil count correlated with disease activity [28]. We could not find a clear correlation between tumor mass and absolute granulocyte count in the reported patient.

Symptomatic paraneoplastic eosinophilia could be treated with drugs leading to decreased production and function of eosinophilic granulocytes including glucocorticoids, hydroxyurea, vincristine [29,30]. Furthermore, reduction of tumor mass either by surgery or systemic treatment has been shown to reduce peripheral eosinophilic counts in paraneoplastic hypereosinophilia [11,28].

In the present case, a combination of drugs directly targeting function and production of granulocytes (prednisone, hydroxyurea, vincristin, cytarabine) and drugs targeting renal carcinoma (sunitinib and temsirolimus) led to a decrease of absolute leukocytes and eosinophils. Neurologic impairment and general status significantly improved with reduced numbers of eosonophilic granulocytes. First line sunitinib had to be temporarily replaced by temsirolimus, as swallowing difficulties did not permit oral therapy. Temsirolimus led to a short-term response (according to RECIST) 2 weeks after initiation. However, rapid progression was observed only 4 weeks after initiation of systemic therapy. As reduction of prednisone led to significant hypereosinophilia-associated reduction of vigilance with prompt improvement after increase of dosage we consider prednisone as a mainstay of the therapeutic approach. Hence, it leads to reduced eosinophilic count and improvement of hypereosinophilia associated symptoms.

## Conclusions

This is the first reported case in the literature of excessive paraneoplastic hypereosinophilia in a patient with metastatic renal cell carcinoma. Paraneoplastic hypereosinophilia due to renal cell carcinoma might indicate poor prognosis and rapid disease progression. Myelosuppressive therapy is required in symptomatic patients.

## Consent

Written informed consent was obtained from the next of kin of the patient for publication of this Case report and any accompanying images. A copy of the written consent is available for review by the Editor-in-Chief of this journal.

## Competing interests

The authors declare that they have no competing interests.

## Authors' contributions

TT was responsible for concept, design, acquisition and interpretation of data. SW contributed to concept, design, acquisition and interpretation of data. CHW was responsible for microscopic and histopathologic elements. SH performed analysis and interpretation of cranial imaging. MH was responsible for radiologic elements and interpretation. JH was responsible for cytokine analysis and interpretation of data. AS contributed to concept and design of the study. LK contributed to concept and design of the study. CS was responsible for acquisition of data, interpretation and critical revision of the manuscript. All authors read and approved the final manuscript.

## Pre-publication history

The pre-publication history for this paper can be accessed here:

http://www.biomedcentral.com/1471-2490/12/7/prepub
